# Mechanical and Wear Studies of Boron Nitride-Reinforced Polymer Composites Developed via 3D Printing Technology

**DOI:** 10.3390/polym15224368

**Published:** 2023-11-09

**Authors:** Ramaiah Keshavamurthy, Vijay Tambrallimath, Swetha Patil, Ali A. Rajhi, Alaauldeen A. Duhduh, T. M. Yunus Khan

**Affiliations:** 1 Department of Mechanical Engineering, Dayananda Sagar College of Engineering, Bangalore 560078, India; keshavamurthy.r@gmail.com (R.K.); patilshweta999@gmail.com (S.P.); 2 Department of Automobile Engineering, Dayananda Sagar College of Engineering, Bangalore 560078, India; 3 Department of Mechanical Engineering, College of Engineering, King Khalid University, Abha 62529, Saudi Arabia; arajhi@kku.edu.sa (A.A.R.); alaaduhduh@jazanu.edu.sa (A.A.D.);; 4 Department of Mechanical Engineering Technology, CAIT, Jazan University, Prince Mohammed Street, P.O. Box 114, Jazan 45142, Saudi Arabia; alaaduhduh@jazanu.edu.sa

**Keywords:** fused deposition modeling, boron nitride, polylactic acid, tensile strength, dimensional accuracy, wear

## Abstract

In the realm of 3D printing, polymers serve as fundamental materials offering versatility to cater to a diverse array of final product properties and tailored to the specific needs of the creator. Polymers, as the building blocks of 3D printing, inherently possess certain mechanical and wear properties that may fall short of ideal. To address this limitation, the practice of reinforcing polymer matrices with suitable materials has become a common approach. One such reinforcement material is boron nitride (BN), lauded for its remarkable mechanical attributes. The integration of BN as a reinforcing element has yielded substantial enhancements in the properties of polylactic acid (PLA). The central objective of this research endeavor is the development of polymer composites based on PLA and fortified with boron nitride. This study undertakes the comprehensive exploration of the compatibility and synergy between BN and PLA with a keen focus on examining their resultant properties. To facilitate this, various percentages of boron nitride were incorporated into the PLA matrix, specifically at 5% and 10% by weight. The compounding process involved the blending of PLA and boron nitride followed by the creation of composite filaments measuring 1.75 mm in diameter and optimized for 3D printing. Subsequently, test specimens were meticulously fabricated in adherence with ASTM standards to evaluate the ultimate tensile strength, dimensional accuracy, wear characteristics, and surface roughness. The findings from these assessments were systematically compared to the wear properties and mechanical behavior of PLA composites reinforced with boron nitride and the unreinforced PLA material. This study serves as a foundational resource that offers insights into the feasibility and methodologies of incorporating boron nitride into PLA matrices, paving the way for enhanced polymer composite development.

## 1. Introduction

In recent years, there has been a significant surge in the utilization of materials derived from renewable sources. Biopolymers, as an eco-friendly alternative to petroleum-based polymers, have gained prominence due to their biocompatibility, biodegradability, and renewability [1]. Among the myriad biopolymers available, polylactic acid (PLA) has emerged as a prominent choice. PLA is a thermoplastic aliphatic polyester produced from renewable resources like corn, wheat, cellulose, sugar cane, and starch. It distinguishes itself with its exceptional barrier properties, high strength, biocompatibility, elevated modulus, low toxicity, heat resistance, improved processability, and transparency. PLA finds applications in a wide range of fields, including medicine (porous meshes, scaffolds, drug delivery systems, and implants), automotive, packaging, and even in the creation of urinary bladder scaffolds.

However, the practical utility of these materials has been hindered by their limited thermal stability, rigidity, brittleness, and slow crystallization rate [2]. Nevertheless, the favorable biodegradability and mechanical properties of PLA have spurred extensive research into its production. Numerous endeavors have been undertaken to enhance PLA’s properties by introducing various fillers. The literature demonstrates the use of substances such as clay [3], cellulose crystals [4], natural fibers [5], and graphene [6] as well as oxides, carbides, and nitrides to enhance specific attributes, rendering them suitable for a diverse array of applications. The addition of particles to PLA has proven to be an effective method for altering its physical and mechanical properties [7]. Boron nitride (BN), with its impressive thermal conductivity and mechanical properties, is a potential polymer-reinforcement material. The introduction of BN as a reinforcement for PLA will significantly improve their properties, making them more suitable for electronic product packaging. According to the available literature, PLA/BN composites have never been the subject of exhaustive research [7,8,9]. PLA ranked as the second most consumed natural plastic in the world in 2010, but it is not yet a standard polymer. Multiple physical and processing limitations have hindered the widespread deployment of PLA [10]. Rapid prototyping, also known as additive manufacturing, is a technique for producing prototypes and end-use parts directly from three-dimensional digital data. One of the major benefits of these technologies is the ability to produce personalized products with minimal lead time and expense. In contrast to subtractive and joining techniques used in conventional production systems, additive manufacturing operates by stacking material layer by layer. Feeders or extruders feed filaments or wires into the nozzles and heat them to a liquid or molten state as part of the FDM process. The material is extruded through nozzles when it has been heated slightly above its melting point. The final product consists of layers of molten material deposited in layers and rapidly cooled during deposition [11]. Various studies have investigated various filament types for FFF. Gray et al. [12] made an attempt to develop thermotropic liquid crystalline polymers (TLCPs), which were combined with a polypropylene (PP) matrix to improve the tensile properties of polymer composites so that they could be used as functional components in FFF. A morphological and tensile analysis of filaments extracted via dual extrusion was performed in this study. Zhong et al. [13] conducted a successful study on the use of short reinforcement fiber in an ABS matrix. The composite filament could be used for FFF due to its increased strength. The tensile strength and morphology of the filaments extracted via dual extrusion were analyzed as properties. Shofner et al. [14] manufactured a polymer composite by combining an ABS matrix with single-walled carbon nanotubes. G. Melenka et al. [15] conducted an experiment to develop a polymer composite by adding continuous Kevlar fibers to an ABS matrix. As part of the study, the filler material was altered to determine whether or not the tensile properties varied. The amount of Kevlar fibers added ranged from 4.04 to 8.08 to 10.1%. Along with the strain and Young’s modulus, the material’s tensile strength increased as it was gradually reinforced. Perez et al. [16] analyzed the effect of various filler materials on the tensile strength of composites. ABS was chosen as the matrix material for the preparation of the samples. Separately incorporated thermoplastic elastomer, jute fiber, and TiO_2_ samples were compared to ABS-only samples. ABS-TiO_2_ had a greater tensile strength than jute fiber and TPE, whereas ABS-TiO_2_ had a greater tensile strength than jute fiber and TPE. When two polymers are combined, there will be an improvement in certain properties. The most commonly employed blends are polycarbonate and acrylonitrile butadiene styrene. By combining these materials, superior impact resistance and thermal stability are achieved. As a result of its enhanced mechanical properties and stability [17], it is an excellent material for the polymer industry. Automobile and home appliance manufacturing are major industries that employ these substances [18]. In a fascinating study, additive manufacturing was used for the first time to produce an electrically insulated, thermally conductive material. Using a 3D printing technique, the mechanical and thermal properties of boron nitride (BN)–acrylonitrile butadiene styrene (ABS) composites were investigated. In terms of thermal conductivity, a 45–45° infill had slightly better flexural strength than a 0–90° infill, but the thermal conductivity was insensitive to infill orientation. The mechanical strength and anisotropic heat conductivity of 3D-printed composite materials are frequently diminished. Adding BN flakes modestly increased the flexural modulus [19]. When looking into PLA as a matrix material to be used along with BN as reinforcement, studies related to mechanical and wear characterization are very minimal. Among the other available reviews, natural fiber was added to the PLA matrix in place of synthetic fiber to study heat treatment and hygrothermal ageing. Greater absorption of moisture through the natural fiber played a detrimental role in the reduction in mechanical properties [20]. Using the technique of solvent cast processing, Bindhu et. al. [9] developed a PLA composite reinforced with 4 wt% BN to study the thermal and mechanical properties. In various tests, the tensile strength was noted to increase with the addition of BN as a reinforcement. Tensile strength and wear studies using FDM technology for BN-reinforced PLA have not been carried out by many researchers, and hence this provides a way to explore the desired properties developed using FDM technology. Though 3D printing is an advanced and sought-after technology for manufacturing complex parts with sustainable goals, the challenge lies in the development of a composite filament with desired reinforcement properties and its effective utilization in fabricating a part. Apart from these, a different set of anomalies and defects would arise with a new polymer composite, which needs to be overcome by choosing optimized printing parameters. The most common forms of defects found in printing polymer composites were warping, distortions, voids, uneven reinforcement, and reduced bonding, to mention a few [21]. In another research study, an investigation involving the extrusion and subsequent 3D printing of biocomposite filaments was carried out. These filaments were crafted from poly(lactic acid) and infused with sub-micrometric silicon particles, and the process employed was fused deposition modeling (FDM). Filaments were developed with various weight percentages of silicon particles ranging from 0% to 7%, all with the goal of enhancing the performance of poly(lactic acid) (PLA). The comprehensive analysis involved an in-depth examination of the mechanical and tribological properties of the printed biocomposites using well-established techniques. The findings unveiled notable enhancements in key attributes. For instance, the Shore D hardness and tensile strength reached 55 MPa and 60 MPa, respectively, while the impact strength stood at 5.9 kJ/m^2^ and the compression strength at 11.8 MPa. These values far surpassed those of the unaltered PLA, which exhibited the lowest results. [22]. In yet another study, the primary objective was to enhance the fused deposition modeling (FDM) process parameters for the improved wear performance of polylactic acid (PLA). The research delved into the investigation of key process variables such as layer thickness, orientation, and extruder temperature. Specifically, these parameters were examined to create wear specimens adhering to ASTM G99 standards [22] through the FDM printing process. The primary focus of the study was to analyze the wear characteristics of a polymer pin under the conditions of a low sliding speed. The results of this investigation highlighted the substantial impact of build orientation on the wear performance of the polymer pin [23].

Considering the insights from prior research, our current study is centered on the development of boron nitride (BN)-reinforced poly(lactic acid) (PLA) through the application of fused deposition modeling (FDM). The primary objective of this research is to innovate in the realm of polymer composites with a specific focus on their production via FDM technology. The aim is to address existing limitations, such as issues related to print quality, compounding processes, pore formation, and dispersion concerns.

This article is primarily dedicated to exploring the potential for fabricating specimens that exhibit reduced porosity, optimum extrusion temperatures, and an ideal dispersion of BN within the PLA matrix. To achieve this, we developed filaments suitable for FDM applications by incorporating 5% and 10% by weight of BN into a PLA matrix. Our experimental investigations predominantly concentrated on assessing the tensile and wear characteristics of FDM-produced polymer composites while adhering to ASTM standards.

## 2. Materials and Methods

### 2.1. Polylactic Acid (PLA)

PLA is a common thermoplastic polymer used as a matrix material. PLA plastics were procured in the form of pellets from Adnano Pvt. Ltd., Shivamogga, India. The PLA obtained was of research grade with 99% purity and a molecular weight of 76.12 g/mol. PLA finds large applications in the industrial sector; it is biodegradable in nature and easily processable.

### 2.2. Boron Nitride

**Figure 1:**
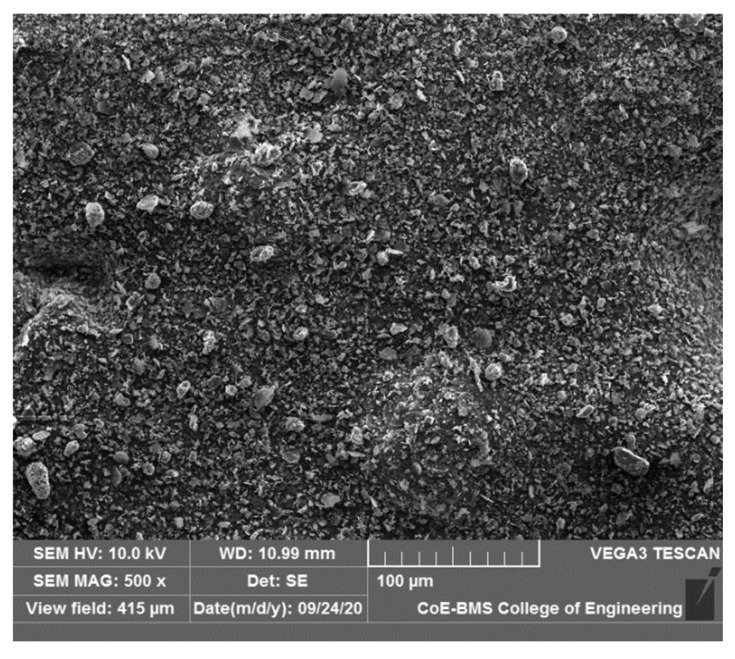
Scanning electron microscopy images of boron nitride.

**Figure 2:**
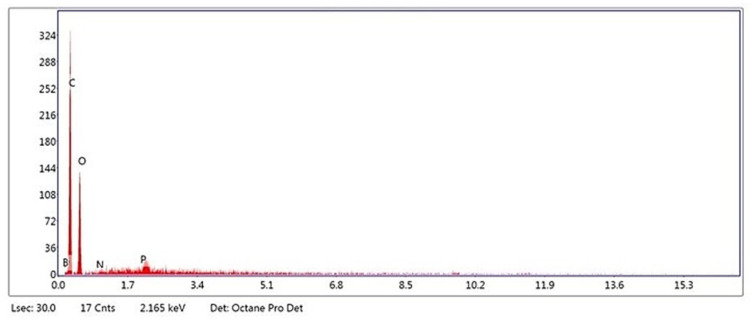
EDAX image of BN.

Fine boron nitride (BN) powder with particle sizes ranging from 40 to 45 μm and 99% purity was purchased from M/s Adnano Pvt. Ltd., India, and was considered for the study. The SEM of the BN is shown in Figure 1, and the EDAX results are shown in Figure 2. A scanning electron microscope (JSM 840a Jeol, Tokyo, Japan) was used for the SEM and EDAX studies at BMS College of Engineering, Bangalore. A sample of the filament was cut using a razor blade to have a flat, planar geometry. A sample was placed on the stub using double-sided tape. The coating of the sample was carried out by spraying Au to make it conspicuous. The images were captured as the final step of the required magnification.

### 2.3. Blending and Extrusion

Boron nitride (BN) powder and PLA pellets were dried in a vacuum oven at a temperature of 80 °C for six hours before the processing began. This was done to remove any absorbed water vapor. In order to combine the various raw materials, a microcompounder with twin screws was utilized. Approximately five minutes were spent compounding the substance at a temperature of 220 °C and 100 rpm. For the purpose of 3D printing, the BN was compounded at weight percentages of 5% and 10% in PLA. In order to create samples that could be 3D-printed, compounds were first extruded from the compounder and then pelletized in a consistent manner. The basic mechanism of the extrusion process comprised a screw that transported the raw plastic pellets from a hopper through a heating zone in a metal pipe where the plastic was melted. The raw plastic pellets were gravity-fed from the hopper into the screw. Inside the pipe, the molten plastic was forced through a die at the end of the pipe to form a filament. The scroll length of the twin screw extruder was maintained at 20 mm. The extruder machine (VFX 500) had a maximum temperature of 350 °C with a mica band heater and 4 zones with independent PID controllers. A variety of polymers could be used in this machine. The temperature measurement system used PT100 sensors with a high sensitivity and reliability. The filament variable diameter was from 0.5 mm to 3 mm, and the screw was composed of a chromium–molybdenum alloy. The pelletized material was extruded into a 3D printing filament by GLS Polymers (Bangalore, India) using a screw extruder. An extruder operating at a temperature of 180 °C and a die with a diameter of 1.75 mm were utilized in this process. The filament had a diameter of 1.75 mm ± 0.05 mm, and it was manually drawn and spooled. A comparable analysis was carried out by Vijay et al. [24].

Before the start of the extraction of the filament, the pellets were dried at a temperature of 120 °C for 2 h. Boron nitride and the dried PLA pellets were mixed in the pre-mixer at a rotor speed of 60 rpm at temperature of 225 °C. Compounding and twin-screw extrusion machines were adopted to extract a filament with a 1.75 mm diameter. Filaments with the addition of 5 and 10 wt% BN were developed. A pure PLA filament was also developed by maintaining an extrusion temperature of 220 °C. The twin extrusion setup was utilized by varying the temperature of various zones for smooth extraction. The developed filaments with a 1.75 mm diameter were used in the fused deposition modeling (FDM) machine.

### 2.4. Fused Deposition Modeling (FDM)

Fused deposition modeling, also known as FDM, is currently one of the most widely used approaches to three-dimensional printing. The development of the 3D model makes use of a CAD file that is saved in STL format. This method can be applied to the production of any complicated component [25]. A Pramaan printer from Global 3D Labs, Karnataka, India, was utilized to fabricate the test specimens for tensile and wear testing. The printer featured an enclosed chamber that had a build volume of 4000 mm^3^. The following criteria served as the basis for the development of the models: an infill density of one hundred percent, a layer thickness of 0.1 mm, a shell thickness of 0.4 mm, bottom and top layer thicknesses of 1.2 mm each, a speed of five millimeters per second, a nozzle orientation of forty-five degrees for PLA, a temperature of 240 °C for fabrication, varying temperatures for other proportions, and a bed temperature of 80 °C. After passing through the nozzle and onto the print bed, the filament moved in the same X, Y, and Z directions as the nozzle did. The X-direction was the direction in which the fabrication of the parts took place. It is generally agreed upon that additive manufacturing is a more environmentally friendly alternative to conventional manufacturing, and it is also acknowledged that this technique helps reduce carbon footprints. In the context of the world as a whole, both the number of case studies and the widespread relevance of their findings have been expanding at an exponential rate [26]. The areas of transportation, construction, aerospace, health care, and electronics are among those in which additive manufacturing (AM) has the potential to have a significant impact on society [27].

### 2.5. Testing and Characterization

The specimen of a dog-bone-shaped bar was produced in accordance with ASTM-D638 [27] for the evaluation of its ultimate tensile strength. Tensile testing was performed on rectangular bar specimens (dog-bone-shaped) according to ASTM standards using the universal testing method (UTM). The testing was carried out at NALRC, Bangalore, India, on an FIE Universal Testing Machine (model number: MV1-PC) with a capacity of 0–6 tonnes.

**Figure 3:**
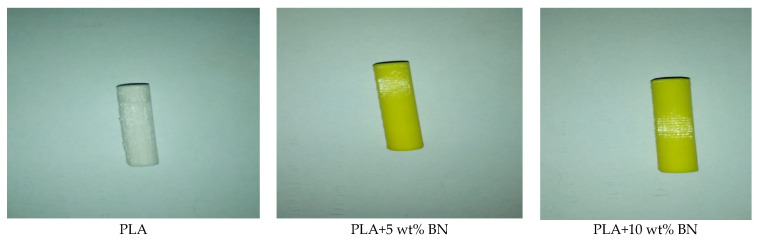
Photographs of wear specimens.

These samples were subjected to surface roughness and dimensional accuracy tests. Three tests were carried out for each set of specimens, and the resultant values were averaged as an arithmetic mean for the surface roughness and dimensional accuracy. Vernier calipers were used to measure the dimensions. A surface tester was used to measure the surface roughness. The developed parts were also subjected to a wear property assessment. Figure 3 depicts photographs of the PLA and PLA + 5 wt% BN wear specimens created with FDM. The instrument used for the wear analysis was a model TR20LPHM-400 manufactured by DUCOM Instruments, Peenya, Bangalore, India. The ASTM G99 test method was adopted to carry out this experiment. The dimensions of the developed parts were a diameter of 8 mm and a length of 20 mm [28].

## 3. Results and Discussion

### 3.1. Microstructure Studies

**Figure 4:**
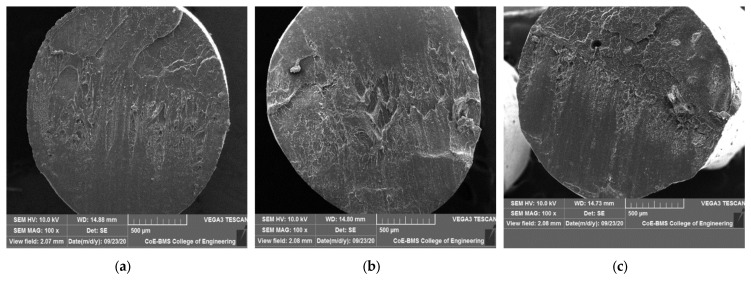
SEM images of (**a**) PLA, (**b**) PLA + 5 wt% BN, and (**c**) PLA + 10 wt% BN.

**Figure 5:**
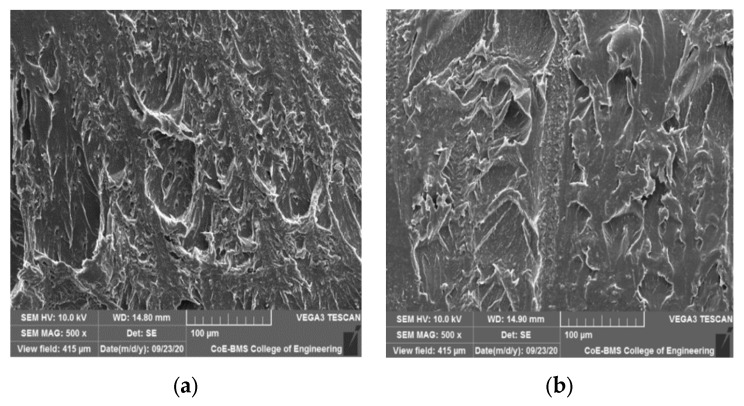
SEM images of BN dispersion: (**a**) PLA + 5 wt% BN; (**b**) PLA + 10 wt% BN.

Figure 4 shows SEM images of PLA, PLA with 5% BN, and PLA with 10% BN. The images show that the filaments reinforced with boron nitride powder had a homogeneous dispersion, demonstrating improved BN wettability in the PLA. Furthermore, there are no visible boron nitride debonding or agglomeration defects in the PLA. The excellent surface-to-area volume ratio of the BN, as well as its general properties, played an important role in demonstrating no aggregation. Isometric dispersion can be seen with equal reinforcement distribution and an irregular shape of the filler material. Higher mechanical properties of the composites were achieved with homogenous dispersion of reinforcement in the matrix. Moreover, porosity and cracks are not visible in the SEM images due to the optimal fabricating parameters. The other major factors that influenced the dispersion characteristics were the wettability, size of the reinforcement particles, processing-time density, heat dissipation, nozzle temperature, layer thickness, and build direction. Joy et al. [28] reported in a study of BN nanocomposites that as the content of BN platelets increased, they interacted with one another and formed a network for transporting heat, greatly improving the material’s ability to conduct heat. The inclusion of BN platelets improved the thermal stability slightly. Because of the good BN platelet dispersion and improved compatibility between the platelets and matrix, the mechanical properties revealed a noticeably higher impact strength at a high BN loading. The electrical insulation properties of the PLA composites were also unaffected by the BN content. Figure 5 shows the SEM images of parts fabricated via FDM.

### 3.2. Surface Roughness

**Figure 6:**
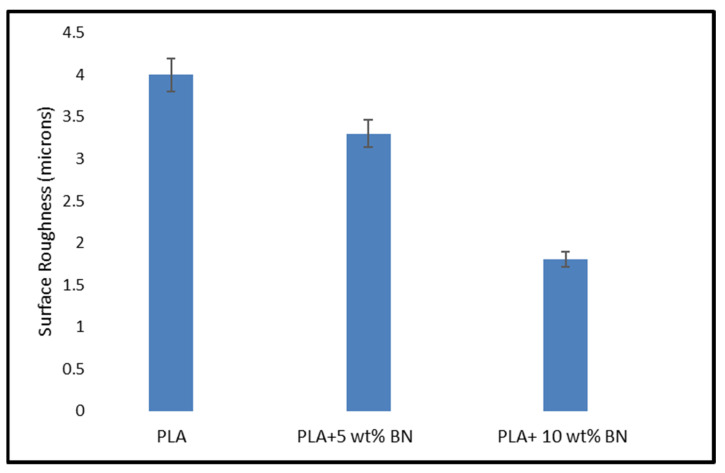
Surface roughness of PLA, PLA + 5 wt% BN composite, and PLA + 10 wt% BN composite.

Figure 6 depicts the surface roughness variations in the FDM components of the PLA polymer and the PLA + 5 wt% BN and PLA + 10 wt% BN composite polymers. The figure shows that as the amount of boron nitride powder was increased, the roughness decreased. The study utilized a model SJ-210 surface tester manufactured by Mitutoyo to measure the surface roughness.

The presence of boron nitride in the composites, in addition to influencing the printing parameters, contributed significantly to the reduction in the surface roughness. This was attributed to several key factors. Firstly, boron nitride exhibited a high thermal conductivity, which played a crucial role in the fused deposition modeling (FDM) process. It enabled more efficient heat dissipation, ensuring a consistent temperature distribution across the printed layers. This effectively minimized the likelihood of thermal inconsistencies that could lead to surface roughness. Furthermore, the incorporation of boron nitride enhanced the flow characteristics of the composite material. This improvement resulted in a more uniform deposition of the material, ultimately leading to smoother surfaces. Additionally, boron nitride particles served as effective fillers within the composite. As their concentration was increased, they adeptly filled gaps and irregularities in the material, contributing to an overall smoother surface texture.

Moreover, the low surface energy and favorable release properties of boron nitride played a significant role in reducing adhesion between the printed layers. This characteristic promoted a smoother surface finish by minimizing the potential for irregularities caused by layer adhesion.

Additionally, boron nitride’s presence could potentially mitigate the material’s tendency to shrink or warp during the cooling phase of the FDM process. This in turn led to a final surface that exhibited significantly reduced irregularities.

The FDM components with boron nitride powder had improved surface finishes, which improved the dimensional stability and uniform mixing. The homogeneity of the boron nitride powder distribution improved the structural stability and bulk thermal conductivity of the FDM components. When compared to unreinforced PLA plastic, the surface roughness of the 5 wt% BN and 10 wt% BN decreased by 13.01% and 24.61%, respectively. Among the various available studies, we observed that the improvement in surface quality was influenced majorly by the build orientation and layer thickness [29,30]. The layered nature of part development can be optimized with lower peaks and valleys by including a minimal layer thickness. An increased nozzle temperature leads to increased fluidity of the filament, causing a roundoff at the raster and hence providing a better surface finish [31].

Regarding post-process treatments such as polishing after fused deposition modeling (FDM), no such treatments were carried out. This is because the objective was to assess the wear resistance of the FDM polymer composite in its as-printed condition without any additional modifications. This approach was valuable in understanding the material’s performance in its raw state, as it would be directly used in practical applications without further processing.

The decision not to polish before wear resistance testing is likely because polishing would alter the surface characteristics, potentially leading to results that do not accurately represent the material’s behavior in its original printed form. However, for all as printed conditions, the surface roughness was recorded without post-printing treatment.

EN 24 steel was used as counterpart material, as it is a widely used engineering steel with good mechanical properties, making it a suitable choice for wear resistance testing. We have not presented details of the counterpart surfaces after testing due to fact that the very minimal damage or identifiable features observed in the steel counterpart disc suggested that the testing conditions may have been more conducive to materials with a lower hardness. This observation further highlighted the relative performance of the FDM polymer composite in comparison to the EN 24 steel under the given testing conditions.

### 3.3. Dimensional Accuracy

**Figure 7:**
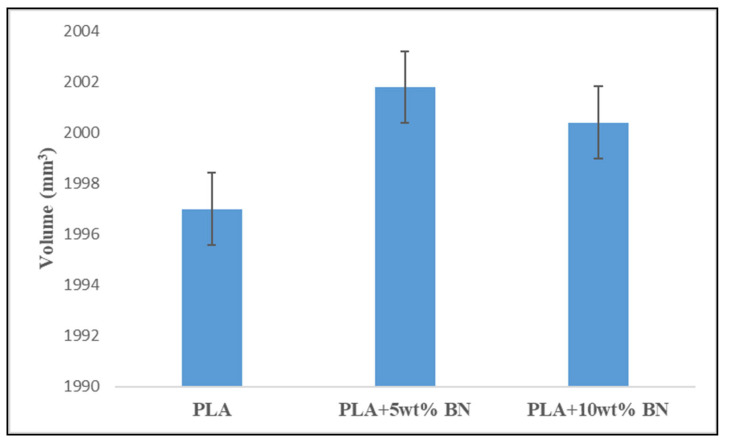
Volume of FDM-processed PLA polymer, PLA + 5 wt% BN composite, and PLA + 10 wt% BN composite with respect to the percentage of reinforcement.

A graph (Figure 7) was plotted for the averaged values of the dimensional accuracy measurements to explain and compare the volumetric shift for the different compositions.

The amount of the dimensional error in PLA plastic can be reduced by increasing the percentage of boron nitride it contains. Components that are constructed using the FDM process benefit from an increased dimensional stability when boron nitride is included in the mix. When compared to the PLA, the dimensional error was found to decrease by 1.041% when BN was added to the PLA at a weight percentage of 5%, and it decreased by 0.494 percent when BN was added at a weight percentage of 10% to the PLA when compared to the PLA + 5 wt% BN. Boron nitride’s lower thermal expansion coefficient and higher thermal conductivity both contributed to the fact that its addition resulted in a reduction in the dimensional error. A reduction in the dimensional error could also be attributed to the optimal process parameters, especially an increase in the bond strength between the layers. The layer thickness, nozzle temperature, enclosed printing chamber, and heat transfer capability also majorly determined the dimensional stability of the printed parts [32,33].

### 3.4. Ultimate Tensile Strength

**Figure 8:**
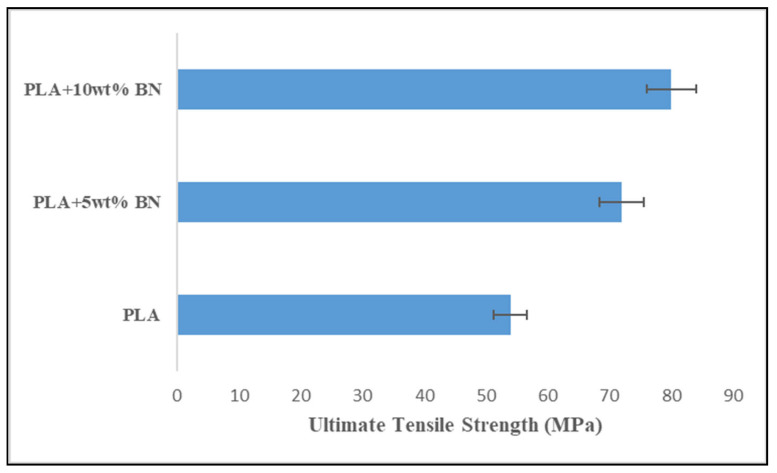
Graph of ultimate tensile strengths.

**Figure 9:**
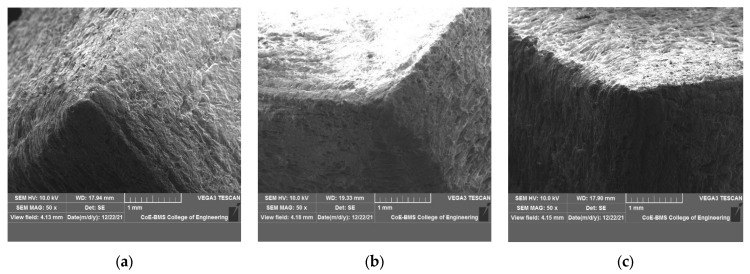
SEM images of specimens fabricated via FDM: (**a**) PLA; (**b**) PLA + 5 wt% BN; (**c**) PLA + 10 wt% BN.

The increased tensile strength of PLA that occurs as a result of the addition of boron nitride is depicted in Figure 8. According to the findings, the ultimate tensile strength of the PLA was significantly improved when the boron nitride powder was included in the manufacturing process. A further observation was that as the percentage of boron nitride in the material increased, the amount of load required for fracture as well as the values for the ultimate tensile strength increased as well. PLA combined with 10 percent BN produced 82 MPa of UTS, while PLA combined with 5 percent BN produced 74 MPa of UTS. In the PLA plastic, increases of 35.504 percent and 46.334 percent were observed after the addition of 5 weight percent and 10 weight percent of boron nitride, respectively. Although pores existed in the parts of the FDM, the specimens showed incremental values of tensile strength with the addition of BN. This was attributed to the molecular organization of the polymer chains through the process of FDM [30]. It has been demonstrated that the fillers, which have mechanical properties that are inherently beneficial, improve the tensile stress performance of composites by acting as a skeletal support structure for the composite [31]. This is because the fillers have mechanical properties that are intrinsically advantageous. By reducing the amount of stress that is concentrated at the interface between the filler and the matrix, a limited or nonexistent agglomeration in the composites helps to prevent stress transfer failure in continuous polymer phases [32]. In addition, the increasing surface contact region between the matrix and the filler makes it possible for the force to be transferred to the network of BN in an efficient manner, which contributes to the improvement in the mechanical strength of such composites [32]. The researchers Zhao et al. [33] investigated what happened when BN was mixed in with aramid fibers. Both the in-plane thermal conductivity and the tensile strength of the multilayer gradient BN/ANF films were found to be significantly higher than average. Voids and interfaces were bound to occur in the FDM-printed parts. The SEM images shown previously indicate indivisible voids in the pure PLA. The addition of reinforcement reduced interfaces and voids, leading to enhanced mechanical properties. This can be clearly seen in Figure 9. The processes of compounding and FDM printing helped in the void reduction and the nonformation of agglomerates, leading to an enhanced ultimate tensile strength [34]. The enhanced mechanical strength of the BN-reinforced composites was also due to the alleviated contact surface area between the matrix and filler, which permitted the load to be effectively transferred to the BN network [35]. The process of building a part through an infill pattern also played an important role in determining the strength of the parts. An infill angle of 90° provided better results during orientation in the load direction than 45° did. Similar results were noted by Li et al. [36].

### 3.5. Wear Test

#### 3.5.1. Constant Load

**Figure 10:**
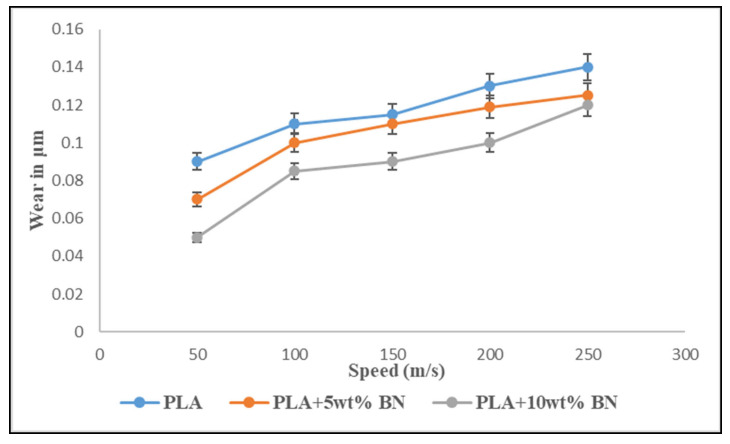
Wear graph of variations in speed while keeping the load constant (10N).

The relationship between the sliding velocity and wear rate of the FDM parts is illustrated in Figure 10. We observed that the rate of wear on the FDM parts accelerated with an increasing sliding velocity, and this trend held true across all of the investigated compositions. When compared to FDM parts that were not reinforced with a boron nitride composite, however, the FDM parts reinforced with a boron nitride composite showed a significant reduction in wear when subjected to the same testing conditions and at the same sliding velocities. At a rotational speed of 250 rpm, the PLA exhibited the highest wear rate with a value of 0.14 m. At the same rpm, the wear rate for PLA mixed with 5 weight percent BN was measured at 0.12 microns, while the wear rate for PLA mixed with 10 weight percent BN was measured at 0.10 microns. The higher temperature at the interface may have been the result of increased wear brought on by an increase in the sliding velocity. The increased temperature caused the surface of the test samples to become more pliable, which led to an increase in wear.

#### 3.5.2. Constant Speed

**Figure 11:**
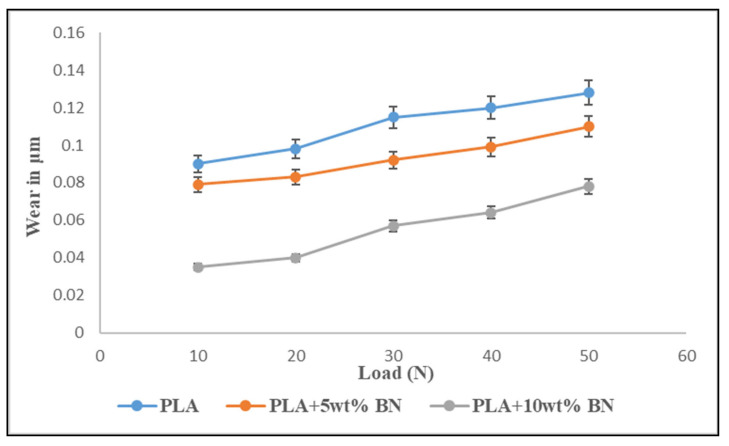
Wear graph of variations in load while keeping the speed constant (50 rpm).

Figure 11 illustrates the impact that changing the load had on the amount of wear that occurred on different FDM component compositions. When the amount of load that was being applied was steadily raised, we observed that the rate of wear increased regardless of the composition being investigated. The PLA + 10 wt% BN showed a greater capacity for bearing loads by exhibiting a wear rate of 0.06 μm per 50 Newtons of pressure. When subjected to the same loads, the PLA + 5 wt% BN showed a wear rate of 0.10 μm, while the PLA showed 0.12 μm. Plastic deformation was greater at higher loads, which can be attributed to the increase in wear that occurred in conjunction with the increase in load. A greater amount of plastic deformation led to the surface of the test sample cracking, which in turn led to a greater amount of material being lost. Additionally, when greater loads were applied, the transfer film or interfacial film that already existed at the interface between the steel disc and the test sample may have become disturbed and unsteady. This opened the door for the actual surfaces to come into contact with one another, which ultimately resulted in an increase in wear. According to the results of all of the tests, the FDM parts reinforced with boron nitride powder showed significantly less wear than the unreinforced ones.

With an incremental load, all three specimens exhibited a similar trend of enhanced wear. Polymers are bound to undergo plastic deformation, and hence this phenomenon was attributed to this process. Deformation led to cracking of the surface, resulting in greater material removal. The interfacial film diluted due to the increased load between the part and disc, resulting in actual physical contact and greater wear [37,38].

#### 3.5.3. SEM of Worn-Out Surface

Amonton’s law [39] can be used to calculate friction, which is the force preventing two surfaces from sliding against one another:

F = μW(1)

where the coefficient of friction is denoted as µ, the normal load (N) as ‘W’, and the friction force as ‘N’. As far as tribology is concerned, µ is a significant factor in determining a component’s wear rate. COF is mainly determined by the characteristics of the surface and lubrication conditions, aside from the nature of the materials [39,40]. It is therefore possible to optimize component performance by tailoring the surface characteristics and lubrication condition [41,42].

**Figure 12:**
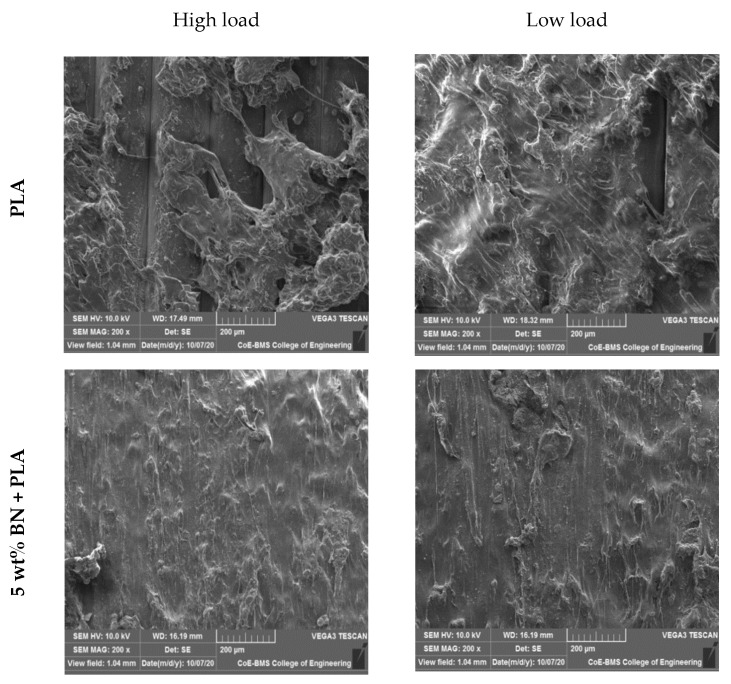

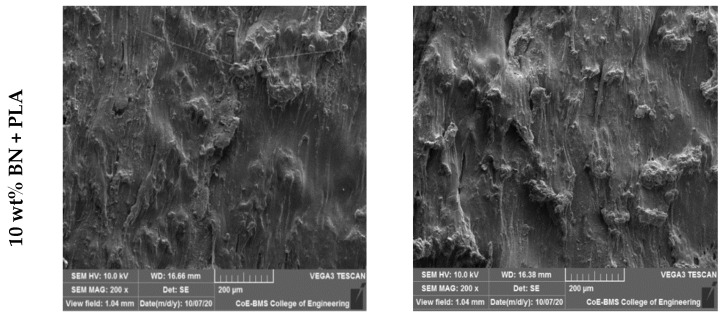
Wear tracks of specimens while keeping the load constant.

**Figure 13:**
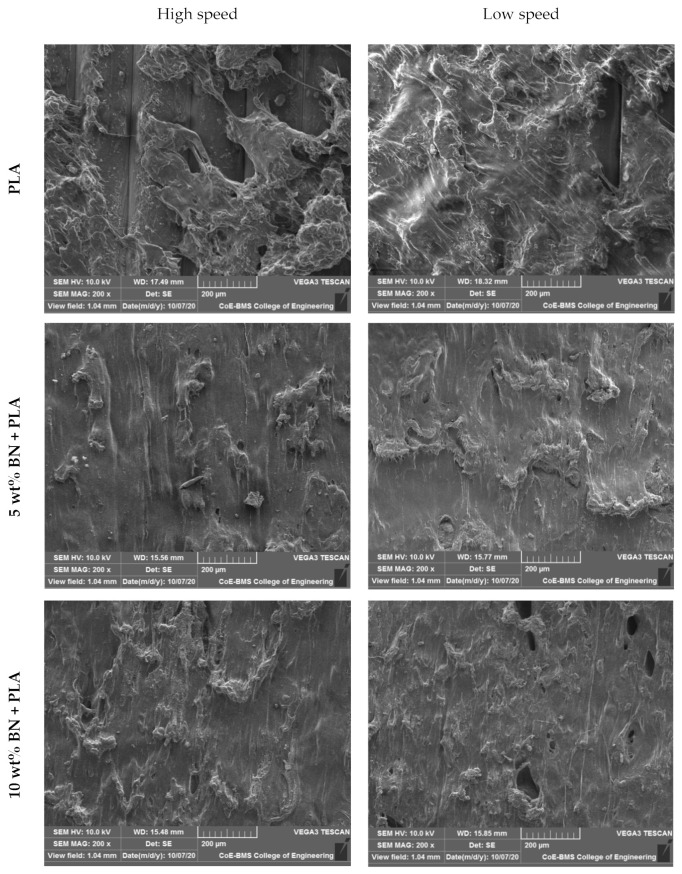
Wear track of specimens while keeping the speed constant.

The wear tracks of pure PLA and boron nitride-filled PLA parts tested with constant loads and a constant sliding velocity are shown in Figure 12 and Figure 13, respectively. We observed that when increasing the sliding velocity and load of the pure PLA and the boron nitride-filled PLA, the boron nitride-filled PLA demonstrated damage on the surfaces, and the extent of the damage increased as the sliding velocity and load were increased. The boron nitride-filled FDM parts, in contrast to the pure PLA parts, exhibited astonishingly low levels of surface damage under all loads and sliding velocities that were investigated. When dealing with the pure PLA, a severe plastic deformation could be observed. The PLA components that were filled with boron nitride did not show any signs of visible surface damage or cracks. This is because boron nitride has improved physical and mechanical properties. Both the increased surface hardness and the improved lubrication characteristics were responsible for the significant reduction in wear loss that was observed. The tribological pattern that was observed through experimentation was demonstrably supported by the surface morphology of the noted surfaces.

The X build orientation of the samples that were developed for the study played an important role in determining the wear behavior. The worn surface of the sample printed in the X orientation had a rough wear pattern and longitudinal lines. The lines, also known as longitudinal lines, were created when the printing layers were applied. The presence of such a structure had the potential to facilitate the distribution of loads across a larger sliding contact area, thereby lowering the wear rate. The tribological characteristics of components that have been 3D printed are significantly influenced by the orientation of the printing process as well as the printing direction. It is possible that the matrix’s increased wear resistance and decreased coefficient of friction were the result of its uniform grain distribution, which could be found throughout the matrix. Because of this uniform dispersion between the PLA printed layers, the final product was a structure that was not only harder and more long-lasting but also more resistant to wear. The friction force could be controlled in the end by the internal lubricator that developed as a consequence of the low concentration of BN loading.

The most common type of wear is called adhesive wear, and it causes the formation of a significant number of lamellar fragments during the wear process of pure PLA. Additionally, the surface of a PLA matrix is frequently damaged as a result of the lamellar fragments. Because of this, pure PLA has poor properties when it comes to abrasion resistance. When BN was added to the polymer matrix, the surface fragments became smaller, and the polymer surface sustained less damage; as a result, the wear rate of the composite was also reduced. The typical abrasive wear seen on composite surfaces could be identified by the light scuffing and ploughing that occurred across the surface that had been worn. This reinforcing effect was primarily caused by a number of key factors, the most important of which were a high aspect ratio and a high strength of the BN. In PLA matrices that had well-dispersed BN, the polymer molecules were able to interact with the BN surface area over a large area, which helped to facilitate an efficient load transfer to the BN network and suppressed the formation of more wear debris in addition to providing protection against frictional forces (thin transfer films formed as uniform). All of these factors contributed to the increased load-bearing capacities and higher wear resistance of the PLA composites. In light of this finding, it should come as no surprise that the significant anti-wear effect exhibited by the filler in graphene-modified polymer composites [43,44] is in agreement with the aforementioned conclusion. There is a possibility that the loadings of BN in a PLA matrix reach saturation for nano-composites with a higher BN content (greater than 10 wt%) and that there are some aggregates of BN in these materials. According to the findings, BN aggregates have the potential to act as stress concentration points and reduce the load transmission between the polymer matrix and the BN. As a direct consequence of this, the level of surface damage on the nanocomposite increases significantly, the wear resistance begins a precipitous decline, and a great number of large damage pits begin to appear.

## 4. Conclusions

Components of PLA and PLA reinforced with boron nitride developed using FDM were subjected to a comparative analysis that proved the provision of enhanced mechanical and wear properties. Using the twin-screw extrusion method, boron nitride-filled PLA filaments were synthesized successfully up to a 10 wt% BN addition. As the percentage of boron nitride increased, the surface roughness and dimensional accuracy of the FDM components improved, providing a positive outcome of desired results. With the addition of 5 and 10% by weight of boron nitride, the ultimate tensile strength of the FDM components was significantly enhanced, showing appropriate dispersion and enhanced bonding. Compared to the unreinforced PLA plastic, the FDM components reinforced with boron nitride possessed superior wear resistance. There was a tendency for the wear rate to increase as the applied load and sliding velocity increased for each of the tested compositions. Boron nitride-filled FDM components have superior mechanical, tribological, and physical properties to FDM components without reinforcement. The scope for utilization of functional BN, a higher percentage of BN, and post-processing of FDM parts remain the future objectives of our research.

## Data Availability

The data are not available.
